# The Risk of Osteoporosis and Osteoporotic Fracture Following the Use of Irritable Bowel Syndrome Medical Treatment: An Analysis Using the OMOP CDM Database

**DOI:** 10.3390/jcm10092044

**Published:** 2021-05-10

**Authors:** Gyu Lee Kim, Yu Hyeon Yi, Hye Rim Hwang, Jinmi Kim, Youngmin Park, Yun Jin Kim, Jeong Gyu Lee, Young Jin Tak, Seung Hun Lee, Sang Yeoup Lee, Youn Hye Cho, Eun Ju Park, Youngin Lee

**Affiliations:** 1 Department of Family Medicine, Medical Research Institute and Busan Tobacco Control Center, Pusan National University Hospital, Busan 49241, Korea; happygaru@hanmail.net (G.L.K.); hezera83@naver.com (H.R.H.); yujkim@pusan.ac.kr (Y.J.K.); eltidine@hanmail.net (J.G.L.); 03141998@hanmail.net (Y.J.T.); greatseunghun@daum.net (S.H.L.); 2 Pusan National University School of Medicine, Yangsan 50612, Korea; saylee@pnu.edu (S.Y.L.); younghye82@naver.com (Y.H.C.); everblue124@hanmail.net (E.J.P.); ylee23@daum.net (Y.L.); 3 Clinical Trial Center, Department of Biostatistics, Biomedical Research Institute, Pusan National University Hospital, Busan 49241, Korea; jinmi@pusan.ac.kr; 4 Department of Family Medicine, National Health Insurance Service Ilsan Hospital, Goyang 10444, Korea; davidympark@gmail.com; 5 Family Medicine Clinic, Obesity, Metabolism and Nutrition Center Pusan National University Yangsan Hospital, Yangsan 50612, Korea

**Keywords:** irritable bowel syndrome, osteoporosis, treatment for IBS

## Abstract

Patients with irritable bowel syndrome (IBS) are at increased risk of osteoporosis and osteoporotic fracture. This study investigated whether IBS medication attenuated the rate of osteoporosis and osteoporotic fracture risk. We conducted a retrospective large-scale multicenter study across eight hospital databases encoded in the Observational Medical Outcomes Partnership (OMOP) Common Data Model (CDM). The primary outcome was the incidence of osteoporosis, whereas secondary outcomes were osteoporotic fractures. After 1:4 matching, 24,723 IBS patients, 78,318 non-IBS patients, 427,640 non-IBS patients with IBS medication, and 827,954 non-IBS patients without IBS medication were selected. The risk of osteoporosis was significantly increased in the IBS group compared to the non-IBS group (hazard ratio (HR) 1.33; confidence interval (CI) 1.17~1.51). Even in patients who were not diagnosed with IBS, the risk of osteoporosis was significantly increased in those with IBS medication compared to those without (HR 1.77, CI 1.62~1.93). The risk of osteoporotic fracture was significantly increased in the IBS medication group (HR 1.69, CI 1.55~1.84). Patients exposed to IBS treatment even without IBS diagnosis were at increased risk of osteoporosis and osteoporotic fracture. Early diagnosis and treatment of osteoporosis should be considered in patients who have received medication for IBS symptoms.

## 1. Introduction

Recent studies have suggested that irritable bowel syndrome (IBS) could be a risk factor for osteoporosis, although the pathogenesis is still unknown. A meta-analysis of five studies investigated the risk of osteoporosis among patients with IBS, and the pooled analysis found that patients with IBS had a significantly higher risk of osteoporosis than individuals without IBS [1]. Chronic inflammatory conditions, hyperactivity of the hypothalamic pituitary adrenal gland, and nutritional deficiencies have all been implicated in a high risk of developing osteoporosis [2]. Chronic inflammatory conditions in IBS patients are known to increase intestinal irritability due to mucosal inflammation and have an additional effect on gastrointestinal absorption [3]. Osteoporosis is considered to be a multifactorial systemic disease [4]. It is known that chronic inflammatory pathophysiology that affects gastrointestinal absorption is commonly associated with osteoporosis [5].

The cause and pathophysiology of IBS is unclear, and the symptoms experienced by each patient are diverse, thus, there is no single treatment commonly applied for the IBS subtypes [6]. Dysregulation of the intestinal autonomic nervous system is associated with changes in bowel habits. Beginning with a change in emotional state or environment, the bidirectional interaction between the cerebral cortex-limbic system and the gastrointestinal tract may cause gastrointestinal symptoms such as abdominal pain and discomfort, known as “gut-brain axis” [7,8].

Symptom management is the mainstay of IBS treatment, and care is ideally personalized based on the predominant symptom [9]. Nonpharmacological interventions include dietary and lifestyle modifications, which are generally considered basic management [10]. Pharmacologic therapies are directed at reducing symptoms, such as constipation, diarrhea, bloating, or pain. The type, number, and duration of medications used depend on the symptom severity and response of individual patients. Patients with IBS who do not respond to lifestyle and diet modifications often seek medical attention. These patients could be regarded as having a more severe disease stage. According to a recent study in Korea, 87.6% of patients with IBS who visited a hospital received a prescription [11]. There have been reports that medications, such as proton pump inhibitors (PPI) and selective serotonin receptor inhibitors (SSRI), which are used as treatments for IBS, could cause osteoporosis [12].

We hypothesized that medical treatment for IBS could increase the risk of osteoporosis and osteoporotic fracture. Therefore, we aimed to compare the risk of new onset osteoporosis and fracture in those exposed to IBS medication in the IBS and non-IBS cohorts and investigated the risk of osteoporosis and fracture in the non-IBS cohort according to IBS medication.

## 2. Materials and Methods

### 2.1. Network and Tools

In this study, we used the distributed research network of the Observational Health Data Sciences and Informatics (OHDSI) collaborative [13] to conduct a multicenter retrospective cohort study including eight hospital databases investigating the risk of osteoporosis and fracture between the target and comparative cohorts. To reduce the influence of confounders from observational studies, we applied analysis methods, such as propensity score (PS) adjustment and 1:4 matching, and negative control that could be used in the ATLAS tool (Copyright © 2021 Observational Health Data Sciences and Informatics; https://ohdsi.org/analytic-tools/ (accessed on 26 March 2020)), an analysis tool of OHDSI [14], to quantify and adjust for residual unmeared bias. The Observational Medical Outcomes Partnership (OMOP) Common Data Model (CDM) is designed to enable systematic analysis even with different observational databases [15]. The reason for this is that the data contained in different databases were converted into a standardized data structure through data extraction-transformation-loading (ETL), and then the structure of the data is also converted into a common format (CDM). This is because a systematic analysis is performed using a library of analysis routines.

The OMOP CDM contains 39 tables, which refer to standardized vocabularies, standardized clinical data, standardized health economics, standardized health system data, standardized meta-data, and standardized derived elements [16].

After data from the individual institutions are entered into the OMOP CDM database, various hypotheses can be tested using standardized analytics tools. The ETL tools (evidnet, Gyeonggi-do, Republic of Korea) and the data analysis tool (ACHILLES, PLATO et al.) were created for data quality and characterization, comparative effectiveness, and patient-level predictive modeling [17].

### 2.2. Data Source and Study Population

We conducted a retrospective study that included eight hospital data sources, encoded in the OMOP CDM version 5, from the participating distributed research network, which includes the OHDSI community [18]. The data sources included eight hospitals located in different regions.

To use as many data as possible, we applied different observation periods for each institution. All data sources are claims records: Ajou University Hospital (AUMC, 2.7 million (M); January 1994~May 2020), Daegu Catholic University Hospital (DCMC, 1.7 M; December 2018~January 2005), Gangdong Sacred Heart Hospital (KDH, 1.1 M; October 1986~December 2019), Kangdong Kyunghee University Hospital (KHNMC, 0.74 M; January 2006~December 2019), Kangwon National University Hospital (KWMC, 0.54 M; January 2003~September 2018), National Health Insurance Ilsan Hospital (NHIMC, 1.4 M; June 2018~January 2003), Pusan National University Hospital (PNUH, 0.79 M; February 2011~August 2018), and Wonkwang University Hospital (WKUH, 0.8 M; March 1998~May 2020).

All were mapped to the OMOP CDM schema, providing a uniform format for healthcare data and standardization of underlying clinical disease coding systems; thus, analysis could be carried out by sharing analysis codes across the research network [19]. The OHDSI network studies were performed through a CDM, where access to de-identified patient information and statistical analysis were enabled inside the firewall of the research network; therefore, we collected aggregate results minus the patient-level information for meta-analysis. The entire analytical process was pre-specified before execution, ensuring uniformity in study designs across databases. Our study design was approved by the Institutional Review Board of Pusan National University Hospital (approval ID: 2001-002-086) and the informed consent requirement was waived due to anonymity of the data and the retrospective nature of the study.

Patients <19 years of age with a history of abdominal and/or gastrointestinal disease, on osteoporosis-causing medication, or with a history of osteoporosis were excluded from this study. The list of excluded diseases and concept IDs is presented in the Supplement Materials.

The IBS cohort was defined as patients ≥19 years of age with IBS who were receiving IBS treatment. We defined the index date in the IBS cohort as the first date a person was diagnosed with an IBS diagnostic code such as concept ID 75576, including descendants with a three-month wash out. Only patients enrolled in the database for continuous observation at least 90 days prior and 0 days after the event index date were included; the initial events were limited to the earliest event per person. The non-IBS cohort was defined as patients with any condition except for IBS, who met the exclusion criteria described in Table S1, and were enrolled in the database for continuous observation at least 90 days prior and 0 days after the event index date; the initial events were limited to earliest event.

We defined the time-at-risk to start on the day of the cohort start date, and stopped 3650 days from the cohort start date, allowing for the minimum number of days at risk of one.

### 2.3. Exposure

We identified IBS medical treatments using the care recommendation for patients with irritable bowel syndrome [9]. The initial exposure occurred when a medication of interest was prescribed. Continuous medication exposures were defined by allowing fewer than 30-day gaps between prescriptions. We used OHDSI’s large, diverse population to characterize treatment pathways constructed here as the order of medication use by the patient as prescribed. In each hospital data analysis, the treatment pathways prescribed in the IBS and non-IBS groups could be compared through a sunburst plot. The list of concept IDs and medications used to treat (1) diarrhea, (2) constipation, (3) pain, and (4) microbiota were as follows: (1) 948555 alosetron, 1501617 colestipol, 43013047 crofelemer, 930916 diphenoxylate, 46234135 eluxadoline, 991876 loperamide, 1000560 ondansetron; (2) 940426 calcium polycarbophil, 949279 carboxymethylcellulose, 42900505 linaclotide, 987366 lubiprostone, 993631 magnesium oxide, 1592897 plecanatide, 986417 polyethylene glycol 3350, 957797 psyllium, 759740 pyridostigmine, 916943 tegaserod; (3) 710062 amitriptyline, 40234201 butylscopolamine, 997276 cimetidine, 716968 desipramine, 924724 dicyclomine, 19056611 drotaverin, 715939 escitalopram, 755695 fluoxetine, 751412 fluvoxamine, 778268 imipramine, 19008994 mebeverine, 19080226 milnacipran, 19016099 octylonium, 722031 paroxetine, 19086712 peppermint oil, 19025198 pinaverium, 739138 sertraline, 743670 venlafaxine, 40234834 vilazodone, 40799195 zimeldine; (4) 43009037 bacillus licheniformis, 42898675 bacillus subtilis, 44012535 beta-galactosidase, 794109 Enterococcus faecium, 987153 Lactobacillus acidophilus, 45775207 Lactobacillus casei, 19000811 Lactobacillus casei rhamnosus, 19122437 Lactobacillus rhamnosus GG, 43008987 Lactobacillus rhamnosus R0011, 1735947 rifaximin, 991855 Saccharomyces boulardii.

### 2.4. Outcomes

For new-onset osteoporosis, patients who had a previous history of osteoporosis or who received osteoporosis medication were excluded with a 3-month washout before the index date. New onset osteoporosis was defined as recently diagnosed osteoporosis or the initiation of osteoporosis medication after the index date. The concept IDs of osteoporosis included 80502 osteoporosis, 417333 primary osteoporosis, and 4010333 postmenopausal osteoporosis. We excluded secondary osteoporosis (concept ID 45766159). The list of osteoporosis medications and concept IDs are as follows: 40222444 denosumab, 44506794 bazedoxifene, 1557272 alendronate, 1512480 ibandronate, 1511646 pamidronate, 1516800 risedronate, 1513103 raloxifene, 1521987 teriparatide, and 1524674 zoledronic acid.

For new-onset osteoporotic fracture, patients who had a previous history of osteoporotic fracture were excluded with a 3-month washout before the index date. New onset osteoporotic fracture was defined as recently diagnosed osteoporotic fracture after the index date [20]. The concept IDs of osteoporotic fracture include hip 4133012, 442560, 45763653, 4230399, and 435093; spine 4009296, 4008356, 4008355, 4053828, 4013613, 4129394, 764899, 764679, 44783966, 764905, 764678, 4140300, 46270349, 4328823, 4209549, 4013596, 4170742, and 437993; proximal humerus 4009431 and 440230; distal radius 4138301, 4134322, 437116, and 40491988.

### 2.5. Statistical Analysis

In each data source, the incidence of osteoporosis and fracture between the target and comparator cohorts were compared. A 1:4 PS [21] matching with a caliper of 0.1 was used to reduce the differences in baseline characteristics, including age and sex, between the two groups. PS was estimated using L1-regularized large-scale logistic regression models based on age groups, sex, index year, condition group, medication group other than IBS treatment medication, Charlson comorbidity index with the L1 penalty hyper-parameter used through 10-fold cross-validation with high-performance computing [22]. The Cox proportional hazards model was used to estimate the relative risk of hazard ratio (HR). Meta-analysis was performed to summarize and quantify the results from the eight data sources using Review Manager version 5.4 (Copyright © 2021 The Cochrane Collaboration; https://training.cochrane.org/online-learning/core-software-cochrane-reviews/revman/revman-5-download (accessed on 26 March 2020 )).

Negative control outcomes were applied for quantification of systematic errors [23]. These negative control outcomes were not thought to be related to medications for IBS, seleted by a data-rich algorithm. A candidate list of negative control outcomes was generated by identifying outcomes with no evidence of being causally related to any exposure of interest [24]. Probable outcomes were presented according to the prevalence of the observational databases, and were selected manually by the researcher as a set of generally accepted negative controls. For further calibration of *p* values for the outcomes, we fit an empirical null distribution to these negative control point estimates to allow further calibration of *p* values [25].

A *p*-value less than 0.05 was judged statistically significant. Statistical analysis was executed within OHDSI’s ATLAS tool version 2.7.6 accessed on March 26, 2020 (Copyright © 2021 Observational Health Data Sciences and Informatics: https://ohdsi.org/analytic-tools/). The entire code used to perform this study can be shared in any database in the format of OMOP CDM to allow analysis by applying the same method used in this study.

## 3. Results

Across all data sources, we identified 30,629 IBS patients and 589,746 non-IBS patients with IBS medication. There were significant differences in age, sex, and Charlson index between the subjects (Table 1).

After 1:4 matching, 24,723 IBS patients and 78,318 non-IBS patients were finally selected. The incidence rate of osteoporosis was increased in the IBS group compared to the non-IBS group (6.57 vs. 4.95 per 1000 person-years). The incidence rate of osteoporotic fracture was increased in the IBS group compared to the non-IBS group (2.33 vs. 1.92 per 1000 person-years) (Table 2).

We identified 589,760 non-IBS patients with IBS medication and 954,159 non-IBS patients without IBS medication for osteoporosis and osteoporotic fracture risk. We performed 1:4 matching and finally 427,640 non-IBS patients with IBS medication and 827,954 non-IBS patients without IBS medication were selected. The incidence of osteoporosis was increased in patients with IBS medication compared to those without IBS medication (5.42 vs. 3.11 per 1000 person-years). The incidence rate of osteoporotic fracture was increased in the IBS group compared to the non-IBS group (2.23 vs. 1.30 per 1000 person-years) (Table 3).

**Figure 1:**
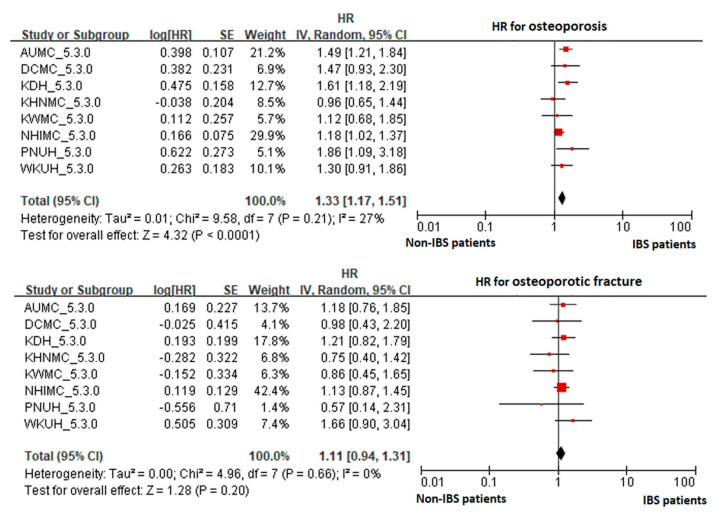
Forest plot of meta-analysis for those who received IBS treatment. Abbreviations: AUMC, Ajou University Hospital; CI, confidence interval; DCMC, Daegu Catholic University Hospital; KDH, Gangdong Sacred Heart Hospital; HR, hazard ratio; IBS, irritable bowel syndrome; KHNMC, Kangdong Kyunghee University Hospital; KWMC, Kangwon National University Hospital; NHIMC, National Health Insurance Ilsan Hospital; PNUH, Pusan National University Hospital; WKUH, Wonkwang University Hospital.

**Figure 2:**
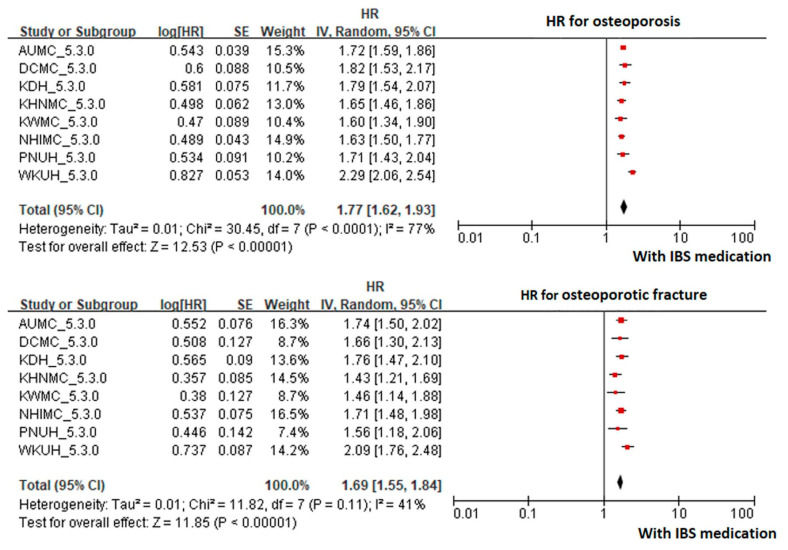
Forest plot of meta-analysis in patients without IBS according to IBS medication. Abbreviations: AUMC, Ajou University Hospital; CI, confidence interval; DCMC, Daegu Catholic University Hospital; KDH, Gangdong Sacred Heart Hospital; HR, hazard ratio; IBS, irritable bowel syndrome; KHNMC, Kangdong Kyunghee University Hospital; KWMC, Kangwon National University Hospital; NHIMC, National Health Insurance Ilsan Hospital; PNUH, Pusan National University Hospital; WKUH, Wonkwang University Hospital.

The risk of osteoporosis was significantly increased in the IBS group compared to the non-IBS group (HR 1.33, CI 1.17~1.51). The risk of osteoporotic fracture was increased in the IBS group compared to the non-IBS group, but the difference was not statistically significant (HR 1.11, CI 0.94~1.31), as can be seen in Figure 1. Even in patients who were not diagnosed with IBS, the risk of osteoporosis was significantly increased when IBS medication was taken compared to when it was not (HR 1.77, CI 1.62~1.93). The risk of osteoporotic fracture was significantly increased in the IBS medication group as seen in Figure 2 (HR 1.69, CI 1.55~1.84).

**Figure 3:**
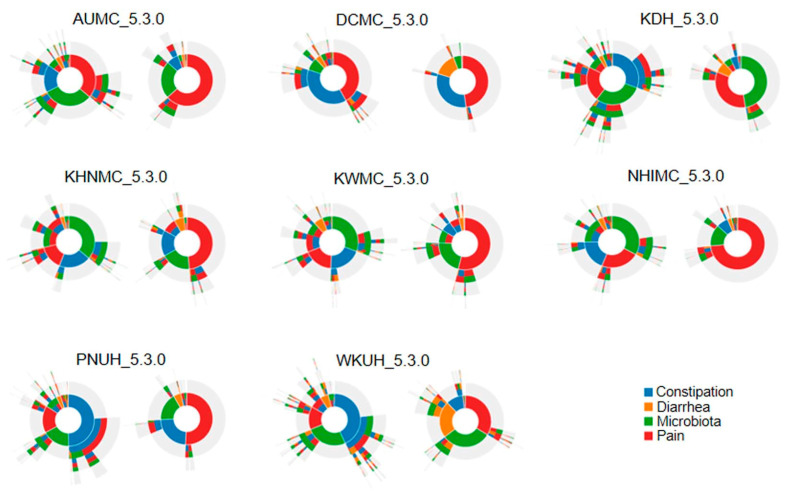
Treatment pathway of selection and use of therapeutic medications in the IBS group on the left and the non-IBS group on the right. Abbreviations: AUMC, Ajou University Hospital; DCMC, Daegu Catholic University Hospital; KDH, Gangdong Sacred Heart Hospital; IBS, irritable bowel syndrome; KHNMC, Kangdong Kyunghee University Hospital; KWMC, Kangwon National University Hospital; NHIMC, National Health Insurance Ilsan Hospital; PNUH, Pusan National University Hospital; WKUH, Wonkwang University Hospital.

There was a difference in the frequency of medication selection priorities in the IBS and non-IBS groups (Figure 3). The pathways revealed that pain, constipation, and microbiota medications were selected first in patients with IBS, and pain medications were selected first in the non-IBS group.

## 4. Discussion

This study is the first study to find that the use of IBS medication is associated with osteoporosis and osteoporotic fractures, using OMOP CDM data. In order to investigate the reason for the increased occurrence of osteoporosis in patients with IBS, subjects were selected considering the use of therapeutic medications to control symptoms. As in previous studies that relied on administrative coding to diagnose IBS and osteoporosis [26,27,28], the summarized HR of osteoporosis was higher in the IBS group than in the non-IBS group (HR 1.33, CI 1.17~1.51). In the IBS group, pain, constipation, and microbiota were widely selected as treatments for symptom control, and pain control medications were often selected in the non-IBS group.

In addition, this is the first study to reveal that osteoporosis and osteoporotic fractures were significantly increased in the patients taking IBS treatment medications; even in those not diagnosed with IBS but receiving IBS treatment, there was a significant increase in osteoporosis (HR 1.77) and fractures (HR 1.69).

Medical treatment for IBS may play a role in the development of osteoporosis. Clinical studies have also shown that prescription of SSRIs and increased risk of osteoporosis and osteoporotic fractures are related [29,30]. SSRIs improve symptoms including gut secretion, peristalsis, intestinal motility, and visceral hypersensitivity [31]. However, bone loss can progress quickly because SSRIs can result in increased levels of gut-derived serotonin, which can then bind to the 5-hydroxytryptamine receptor 1B of osteoblasts and act to inhibit the proliferation of osteoblasts [32]. People with IBS who experience constipation often have lower levels of serotonin; the muscles in their rectums are less reactive to serotonin, and they are more likely to have hard or lumpy stool [33]. When the 5-hydroxytryptamine 3 (5HT3) receptor is activated, it causes contraction of the intestinal muscles. 5HT3 antagonists reduce the depolarization of extrinsic sensory neurons by inhibiting the activation of 5HT3 receptors, and they improve abdominal pain and discomfort because they interfere with signal transfer to the brain [33]. Therefore, serotonin modulators are often used together with laxatives to control symptoms for people with IBS who experience constipation.

Many patients have overlapping symptoms of functional dyspepsia (FD), which includes symptoms of the upper gastrointestinal tract, such as abdominal pain, nausea, heartburn, and indigestion, and IBS, which includes symptoms of the lower gastrointestinal tract, such as diarrhea, constipation, gas, and bloating. According to the symptom analysis, among 354 patients with functional gastrointestinal disorders, 308 were diagnosed with FD, 156 with IBS, and 110 with both symptoms, accounting for 31.1%. Bloating and postprandial distress syndrome were risk factors for IBS-FD overlap [34]. A large number of patients with IBS are not properly diagnosed and can take medication to control symptoms. In addition, medications for controlling FD symptoms sometimes overlap with IBS medications. However, in this study, PPI was not included as a treatment for IBS, because PPI treatment was excluded for the diagnosis of osteoporosis. Therefore, our results may rule out the possibility that PPI use increases the risk of osteoporosis in study subjects.

To date, the understanding of IBS mechanisms has been limited. However, evidence is emerging that microbial factors may be important for IBS pathophysiology. Studies have demonstrated changes in the gut microbiome in patients with IBS. The gut microbiota shows different patterns depending on the type of IBS. Diarrhea-predominant IBS can be diagnosed as positive with a hydrogen breath test because it is accompanied by an overgrowth of intestinal bacteria in the small intestine. In constipation-predominant IBS (C-IBS), methanogenic archaea are often present, thus, C-IBS can be diagnosed as positive with the methane breath test [35]. Dysbiosis is associated with the onset and symptoms of IBS, as in other functional gastrointestinal disorders, such as FD [35]. Qualitative or quantitative changes in the composition of the gut microbiota have revealed a potential role of the gut microbiota in the pathogenesis of gastrointestinal and non-gastrointestinal diseases [36,37]. Gut microbiota disrupts normal intestinal functioning in a variety of ways. Intestinal motility abnormalities, intestinal hypersensitivity, and dysfunction of the mucosal barrier, neuroimmune signaling, and hypothalamus-pituitary-adrenal axis can also occur [38]. Patients with C-IBS harbor more Proteobacteria and Prevotella than healthy individuals. Moreover, patients with C-IBS showed increases in gut microbiota including Alistipes, Desulfovibrio, and Akkermensia. Patients with C-IBS show a reduced presence of Roseburia than healthy individuals [39].

The microbiota plays a role in regulating immune functioning [40], and there are strains among the intestinal microflora that control the immune response and can affect bones, distant organs, and systems [41,42] via the cells of the immune system. Patients with IBS are known to have no visible sign of intestinal inflammation, but researchers have reported that those with IBS have higher levels of cytokines, including interleukin 1 (IL)-1β, tumor necrosis factor, IL-6 and IL-8, resulting in an increased production of serotonin and histamine, which are associated with inflammation [43]. These cytokines directly activate osteoclast production and bone resorption, induce the expression of osteoclastogenic cytokines in osteocytes, and release osteoclastogenic cytokines, which cause osteocyte apoptosis [44].

Nutritional deficiencies in patients with IBS are associated with osteoporosis [45]. There have been studies showing that bile acid malabsorption occurs in patients with IBS [46], which may lead to a lack of micelle formation and impaired absorption of dietary fat and vitamin D, resulting in an increase in vitamin D deficiency compared to the general population [47]. Patients with IBS who have lactose intolerance are advised to avoid milk and dairy products, which may lead to insufficient calcium intake [48]. In addition, a large number of patients with IBS have problems with eating fatty foods [49]. Avoiding fatty foods can lead to intestinal fat malabsorption, which can lead to weight loss or patients becoming underweight, increasing the risk of osteoporosis.

Based on the results of this study, one could consider that IBS and its treatment medication for symptom management could predispose patients to osteoporosis. This association implies that patients with IBS require early evaluation and counseling to prevent the onset of osteoporosis in patients receiving medication for IBS-like symptoms or in patients with other osteoporosis risk factors. Nevertheless, well-designed pathophysiological studies are needed to confirm this association with regards to whether taking medications after a diagnosis of IBS increases the risk of osteoporosis and fractures, compared to those who do not. In addition, it is necessary to investigate whether there is an increased risk of osteoporosis and fracture in subjects experiencing FD as its symptoms may overlap those of IBS.

This study has some limitations that should be noted. Firstly, due to the observational nature of the study, the influence of measured or unmeasured confounders on osteoporosis and fracture incidence cannot be excluded. The data generated from insurance claims may include values that are missing or misclassified before the database is established, and the information related to the patient’s medical history is not always known, resulting in data that are naturally noisy; the use of negative controls in our study is only one attempt to address research bias. Secondly, when relying on diagnostic codes for IBS diagnosis, osteoporosis diagnosis, and fracture definition, it is important to take into account the limitations of diagnosis accuracy and accurate case identification. In addition, the non-IBS group may include patients who have not been diagnosed with IBS but have received medication to control their symptoms. Finally, the database used in this study was anonymized, consequently, it was not known if a patient had visited another medical institution for treatment. Therefore, the same patient may have visited another institution for treatment. However, since each institution is located far away from the others, the occurrence of this situation is less likely.

## 5. Conclusions

In our retrospective, comparative risk, CDM study, we have shown that patients with IBS may have an increased HR of osteoporosis, and even if they are not diagnosed with IBS, taking medications to control IBS-like symptoms may increase the risk of osteoporosis and fracture compared to those who do not take IBS medicine.

## Figures and Tables

**Table 1:** Demographic factors of patients who were prescribed IBS-treatment drugs.

		IBS			Non IBS			*p*-Value
		Count	Mean, SD or %		Count	Mean, SD or %		
Age	AUMC_5.3.0	4555	47.25,	14.42	178,649	44.78,	15.43	<0.001
	DCMC_5.3.0	2012	55.09,	14.83	30,893	49.35,	16.25	<0.001
	KDH_5.3.0	4003	49.45,	16.24	78,039	45.94,	16.54	<0.001
	KHNMC_5.3.0	2323	48.53,	16.10	68,398	50.36,	15.35	<0.001
	KWMC_5.3.0	1276	52.61,	16.93	53,601	47.73,	17.64	<0.001
	NHIMC_5.3.0	10,737	50.26,	16.05	131,941	47.41,	16.28	<0.001
	PNUH_5.3.0	1466	57.79,	14.49	33,587	51.87,	16.81	<0.001
	WKUH_5.3.0	1901	51.37,	15.06	52,063	48.09,	17.27	<0.001
Female	AUMC_5.3.0	2277	50.0		102,909	57.6		<0.001
	DCMC_5.3.0	1142	56.8		19,417	62.9		<0.001
	KDH_5.3.0	2308	57.7		44,015	56.4		0.117
	KHNMC_5.3.0	1288	55.4		41,709	61.0		<0.001
	KWMC_5.3.0	667	52.3		29,055	54.2		0.172
	NHIMC_5.3.0	6046	56.3		79,071	59.9		<0.001
	PNUH_5.3.0	795	54.2		19,558	58.2		0.003
	WKUH_5.3.0	1084	57.0		28,720	55.2		0.108
Charlson comorbidity index	AUMC_5.3.0	721	0.254	1.272	16,894	0.134	0.833	<0.001
	DCMC_5.3.0	493	0.421	1.165	3681	0.178	0.865	<0.001
	KDH_5.3.0	1184	0.439	0.988	6134	0.111	0.740	<0.001
	KHNMC_5.3.0	414	0.267	1.003	5414	0.120	0.895	0.001
	KWMC_5.3.0	391	0.560	1.163	4411	0.125	0.775	<0.001
	NHIMC_5.3.0	1951	0.275	1.026	13,726	0.140	0.719	<0.001
	PNUH_5.3.0	412	0.404	0.973	4540	0.216	1.107	0.001
	WKUH_5.3.0	477	0.400	1.105	6702	0.196	0.890	<0.001

Abbreviations: AUMC, Ajou University Hospital; DCMC, Daegu Catholic University Hospital; KDH, Gangdong Sacred Heart Hospital; IBS, irritable bowel syndrome; KHNMC, Kangdong Kyunghee University Hospital; KWMC, Kangwon National University Hospital; NHIMC, National Health Insurance Ilsan Hospital; PNUH, Pusan National University Hospital; WKUH, Wonkwang University Hospital.

**Table 2:** The risk of osteoporosis and osteoporotic fractures in patients with IBS medication.

	1:4 Matching							
	IBS				Non-IBS			
	Patients	Person-Years	Events	Rate	Patients	Person-Years	Events	Rate
Osteoporosis								
AUMC_5.3.0	4687	25,988	166	6.39	17,636	84,401	366	4.34
DCMC_5.3.0	1701	7445	51	6.85	4619	15,428	68	4.41
KDH_5.3.0	3039	18,096	100	5.53	8999	46,847	155	3.31
KHNMC_5.3.0	2301	8861	49	5.53	7423	23,047	124	5.38
KWMC_5.3.0	1162	5653	37	6.55	3678	13,984	66	4.72
NHIMC_5.3.0	9242	50,562	323	6.39	29,261	135,289	759	5.61
PNUH_5.3.0	1144	4124	35	8.49	3143	10,881	55	5.05
WKUH_5.3.0	1447	8034	85	10.58	3559	15,256	116	7.60
Total	24,723	128,763	846	6.57	78,318	345,133	1709	4.95
Osteoporotic fracture								
AUMC_5.3.0	4687	26,644	33	1.24	17,636	85,715	97	1.13
DCMC_5.3.0	1701	7612	12	1.58	4619	15,582	35	2.25
KDH_5.3.0	3039	18,275	51	2.79	8999	47,029	122	2.59
KHNMC_5.3.0	2301	8951	23	2.57	7423	23,357	65	2.78
KWMC_5.3.0	1162	5725	19	3.32	3678	14,109	41	2.91
NHIMC_5.3.0	9242	51,622	128	2.48	29,261	137,798	258	1.87
PNUH_5.3.0	1144	4221	7	1.66	3143	11,032	21	1.90
WKUH_5.3.0	1447	8303	33	3.97	3559	15,586	33	2.12
Total	24,723	131,353	306	2.33	78,318	350,208	672	1.92

Rate: incidence per 1000 person-years. Matching covariables are age groups, sex, index year, condition group, drug group other than IBS treatment medication, Charlson comorbidity index. Abbreviations: AUMC, Ajou University Hospital; DCMC, Daegu Catholic University Hospital; KDH, Gangdong Sacred Heart Hospital; IBS, irritable bowel syndrome; KHNMC, Kangdong Kyunghee University Hospital; KWMC, Kangwon National University Hospital; NHIMC, National Health Insurance Ilsan Hospital; PNUH, Pusan National University Hospital; WKUH, Wonkwang University Hospital.

**Table 3:** The risk of osteoporosis and osteoporotic fractures in patients without IBS according to IBS medication.

	1:4 Matching							
	with IBS Medication				without IBS Medication			
	Patients	Person-Years	Events	Rate	Patients	Person-Years	Events	Rate
Osteoporosis								
AUMC_5.3.0	130,159	539,339	2779	5.15	242,780	641,442	1860	2.90
DCMC_5.3.0	17,180	68,044	317	4.66	56,505	175,890	473	2.69
KDH_5.3.0	48,861	201,544	751	3.73	101,494	255,885	552	2.16
KHNMC_5.3.0	43,657	156,387	1080	6.91	74,940	159,894	730	4.57
KWMC_5.3.0	32,105	128,286	570	4.44	42,957	108,329	336	3.10
NHIMC_5.3.0	91,027	396,647	2258	5.69	151,062	417,136	1511	3.62
PNUH_5.3.0	24,002	74,428	336	4.51	74,019	174,303	485	2.78
WKUH_5.3.0	40,649	192,960	1442	7.47	84,197	260,602	873	3.35
Total	427,640	1,757,635	9533	5.42	827,954	2,193,481	6820	3.11
Osteoporotic fracture								
AUMC_5.3.0	130,159	548,294	745	1.36	242,780	645,937	511	0.79
DCMC_5.3.0	17,180	68,631	162	2.36	56,505	176,838	253	1.43
KDH_5.3.0	48,861	202,219	571	2.82	101,494	256,277	401	1.56
KHNMC_5.3.0	43,657	158,565	611	3.85	74,940	160,900	445	2.77
KWMC_5.3.0	32,105	129,315	342	2.64	42,957	108,818	192	1.76
NHIMC_5.3.0	91,027	404,082	804	1.99	151,062	420,771	516	1.23
PNUH_5.3.0	24,002	75,107	136	1.81	74,019	175,270	189	1.08
WKUH_5.3.0	40,649	196,736	600	3.05	84,197	262,626	357	1.36
Total	427,640	1,782,949	3971	2.23	827,954	2,207,437	2864	1.30

Rate: incidence per 1000 person-years. Matching covariables are age groups, sex, index year, condition group, drug group other than IBS treatment medication, Charlson comorbidity index. Abbreviations: AUMC, Ajou University Hospital; DCMC, Daegu Catholic University Hospital; KDH, Gangdong Sacred Heart Hospital; IBS, irritable bowel syndrome; KHNMC, Kangdong Kyunghee University Hospital; KWMC, Kangwon National University Hospital; NHIMC, National Health Insurance Ilsan Hospital; PNUH, Pusan National University Hospital; WKUH, Wonkwang University Hospital.

## Data Availability

The data that support the findings of this study are available from the OHDSI study, but restrictions apply to their availability. These data were used under license for the current study, and are not publicly available. The outcome data and codes are, however, available from the authors upon reasonable request, and with permission of the OHDSI study.

## Supplementary Materials

The following are available online at https://www.mdpi.com/article/10.3390/jcm10092044/s1, Table S1: Exclusion criteria for cohort entry.
