# Supplementary material for: The Risk of Osteoporosis and Osteoporotic Fracture Following the Use of Irritable Bowel Syndrome Medical Treatment: An Analysis Using the OMOP CDM Database
Source: J Clin Med. 2021 May 10;10(9):2044. doi: 10.3390/jcm10092044 (PMC8126251; doi:10.3390/jcm10092044)

Supplement 1. Exclusion criteria for cohort entry.

Category	Name	Concept id
**(1) Patients who had a history of abdominal and gastrointestinal disease**		
Neoplastic disease	GI tract malignancy	44501490, 44500731, 44500936, 44499810, 44500363, 44502945, 37018669, 4241792, 4112604, 4243739, 4243114, 4243742, 4243745, 75512, 4241773, 196064, 4241774, 4243878, 4244495, 4243882, 424312, 4244496, 192291, 4244498, 4244501, 4112608, 4244641, 4245560, 4241890, 75210, 4093956, 4244647, 4093955, 200974, 4241922, 36554172, 36683531, 40491001, 443568, 435754, 4095320, 4180790, 411623, 443398, 4092061, 443390, 443381, 443387, 4317409, 40492458,4313503, 442164, 4313517, 436080, 442141, 4317816, 201529, 4232106, 79740, 25748, 197227, 438090, 199747, 80045, 4247719, 193422, 432837, 19, 7500, 441800, 435752, 26638, 4179720, 195482, 4246125, 438699, 74582, 436635, 192836, 196044, 760999, 4247962, 4312022, 200348, 198371, 36539765, 36548501, 4112603, 36536268
	malignant neoplasm of abdomen	4242677, 4242678, 4242683, 45765705, 4243879, 4243125, 4096500, 4241788, 4243887, 192292, 4245553, 4241795, 4241911, 43021774, 197506, 4246127, 443392, 4181328, 4181327, 4180793, 4313511, 36684472, 194589, 40486896, 197806, 197804, 40490929, 201519, 199754, 198091, 4312301, 192568, 198700, 44806773, 4315805, 4315806, 196925, 4314361
	malignant neoplasm of pelvis	4243741, 201238, 201240, 4313916
	malignant neoplasm of endocrine gland	133420, 193144, 4311878
	malignant neoplasm of bone marrow	4247726
	Non-Hodgkin's lymphoma	4038838
	malignant lymphoma	432571
inflammatory disease	ulcerative colitis	81893
	microscopic colitis	4340799
	crohn's disease	201606, 4190185, 4043371
etc.	chronic digestive system disorder	4134593
	celiac disease	4056336, 4241413, 40482828, 194992
**(2) Patients who had a medication that can cause osteoporosis**		
steroid	dexamethasone	42922451, 42922453, 42948533

	triamcinolone	42922375, 42922376, 42941640
	Hydrocortisone	42939642, 42939646, 42939639, 42939631
	methylprednisolone	42934728, 42934742
	prednisolone	42970566
anticancer drug	docetaxel	42922765, 4292231, 41336612, 42921791, 42921543, 41350773
	vincristine	42922339, 42921888, 42939713
	busulfan	42920661
	cytarabine	42920696, 42920697, 42922069, 42920345
	doxorubicin	41361791, 35783409, 35780897, 42946862, 42946876
	vinblastine	42920569
	irinotecan	42920702, 42920500, 42922070, 42922073, 42921304, 42921307
	oxaliplatin	42920581, 42920587, 42920586, 42920230, 42920236, 42920235, 42920059
	cisplatin	42919355, 42920324
	paclitaxel	42920451, 42920133, 42921130, 41358385, 42919743, 42919744
	etoposide	42921410, 42956904
	asparaginase	43268981
	bleomycin	42929924
	cyclophosphamide	1310317, 42944032
	ifosfamide	43294087
	methotrexate	1305058
	mitomycin	21083803, 21034662
	topotecan	42941593
	anastrozole	1348265, 42970739, 1348266
	exemestane	1398399, 1398400
	letrozole	1315946, 1315949, 19022830, 1315947, 42948631, 40058597
	mercaptopurine	42963844
proton pump inhibitor	esomeprazole	42967955, 42968069, 42968036, 41127461, 42968049, 42968161, 42968264, 42968232, 40940024, 42968244

	omeprazole	42945767, 42945673
	ilaprazole	42961064
	lansoprazole	42946137, 42946132, 42946175, 42946164, 42946160, 42946163
	pantoprazole	42929305, 36891946, 43273164, 42929253, 42929258, 42929251, 42970566
	rabeprazole	36886127, 42933802, 42933851, 36882275, 42933642, 42933691
anticonvulsant drug	phenobarbital	42922491, 42956846
	fosphenytoin	42920607, 42921865
	carbamazepine	42919362, 42946835, 42946833, 42946842
	oxcarbazepine	42919272
	valproate	42919003, 42931710, 42931721, 42931727, 42931728, 42931742, 42931743
	levetiracetam	42918821, 42921116, 42935659, 42935670, 42935693, 42935695, 42935723, 42935727
	phenytoin	42921163, 42941522
	ethosuximide	42947177
	gabapentin	42957715, 42957692, 42957703, 42957759, 42957784, 42957674
	pregabalin	42934200, 42934498
	topiramate	42945090, 43274162, 42945058, 42945055, 43295762
antiviral drug	cobicistat	35605556, 21119805
	tenofovir	42708097, 1710312, 35605546, 1718509, 1710281
	elvitegravir	42874212
	emtricitabine	1703093, 35604229, 36268197
GnRH agonist	gonadorelin	42922413
	triptorelin	42922329, 42936716
antidiabetic drug	metformin	42968754
	alogliptin	42970728
etc	heparin	42921076, 42920200, 42921073
	warfarin	42972020, 42972019, 42972015, 42972014
	acetazolamide	42963838

	levothyroxine	42956258, 42956280, 42956272, 42956264, 42956268
	clonazepam	42944760
	furosemide	43861148, 42971383

(3) Patients who had a history that can cause osteoporosis.

neoplastic disease	leukemia	138708, 134305, 135768, 40481524, 140352, 140057, 136056, 134603, 138099, 4038845, 40482847, 317510, 321526, 140666, 133154, 4001331, 135499, 44783718
	lymphoma	4184976, 434592, 4041800, 40486171, 4003830, 432574, 4001328, 4002356, 4003180, 4212994, 4299152, 4147411, 4003833, 4001329, 4002357, 4038841, 432571, 4094544, 440058, 200349, 40481901, 194878, 4038838, 434881, 4294435, 4266805, 40481522, 4301666
	neoplasm of breast, ovary, prostate, and uterus	4162253, 200051, 200962, 45770892
	multiple myeloma	437233
endocrine disease	thyroid disease	4129364, 133729, 138387
	diabetic mellitus	201820, 4063043
	hyperprolactinemia	4030186
	hypopituitarism	4254542
	iatrogenic cushing's disease	4186577
	hyperparathyroidism	136934
autoimmune disease	ankylosing spondylitis	437082
	rheumatoid arthritis	72714, 4253901, 72705, 80809, 4083556, 4035611
	systemic lupus erythematosus	257628
inheritied disease	kleinfelter's syndrome	4228490
	thalassemia	30978
	turner syndrome	4307885
	hemophilia	4236898

condition associated with paralysis	cerebellar stroke syndrome	4111711
	cord injury	4226266, 4235863
	multiple sclerosis	374919
	paraplegia	192606
	tetraplegia	374914
etc	amenorrhea	443800, 4230947
	dementia	4182210, 4314734, 374888, 4046090, 379778, 4218017, 4277444, 4220313, 4278830, 443605
	anorexia nervosa	436675, 4333683
	chronic obstructive lung disease	255573
	congestive heart failure	319835, 314378
	infectious disease associated with AIDS	40493262
	epilepsy	4101747
	sarcoidosis	438688
	major depression	435220

Supplement 2. Subgroup analysis for the risk of IBS treatment on osteoporosis.

Study or Subgroup	log[Hazard Ratio]	SE	Weight	Hazard Ratio IV, Random, 95% CI
**2.1.1 pain**				
AUMC_5.3.0	0.258	0.135	9.7%	1.29 [0.99, 1.69]
DCMC_5.3.0	0.687	0.432	0.9%	1.99 [0.85, 4.64]
KDH_5.3.0	0.252	0.304	1.9%	1.29 [0.71, 2.33]
KHNMC_5.3.0	-0.179	0.305	1.9%	0.84 [0.46, 1.52]
KWMC_5.3.0	0.096	0.318	1.7%	1.10 [0.59, 2.05]
NHIMC_5.3.0	0.305	0.109	14.9%	1.36 [1.10, 1.68]
PNUH_5.3.0	0.58	0.408	1.1%	1.79 [0.80, 3.97]
WKUH_5.3.0	0.504	0.292	2.1%	1.66 [0.93, 2.93]
**Subtotal (95% CI)**			**34.2%**	**1.33 [1.15, 1.53]**
Heterogeneity: Tau² = 0.00; Chi² = 4.70, df = 7 (P = 0.70); I² = 0%				
Test for overall effect: Z = 3.93 (P < 0.0001)				
**2.1.2 microbiota**				
AUMC_5.3.0	0.309	0.152	7.7%	1.36 [1.01, 1.83]
DCMC_5.3.0	0.034	0.659	0.4%	1.03 [0.28, 3.76]
KDH_5.3.0	0.65	0.344	1.5%	1.92 [0.98, 3.76]
KHNMC_5.3.0	-0.231	0.307	1.9%	0.79 [0.43, 1.45]
KWMC_5.3.0	0.447	0.364	1.3%	1.56 [0.77, 3.19]
NHIMC_5.3.0	0.422	0.138	9.3%	1.53 [1.16, 2.00]
PNUH_5.3.0	-0.285	0.628	0.4%	0.75 [0.22, 2.58]
WKUH_5.3.0	0.501	0.31	1.8%	1.65 [0.90, 3.03]
**Subtotal (95% CI)**			**24.3%**	**1.40 [1.19, 1.66]**
Heterogeneity: Tau² = 0.00; Chi² = 6.23, df = 7 (P = 0.51); I² = 0%				
Test for overall effect: Z = 3.96 (P < 0.0001)				
**2.1.3 diarrhea**				
AUMC_5.3.0	-0.179	0.229	3.4%	0.84 [0.53, 1.31]
DCMC_5.3.0	0.663	0.56	0.6%	1.94 [0.65, 5.82]
KDH_5.3.0	0.252	0.528	0.6%	1.29 [0.46, 3.62]
KHNMC_5.3.0	0.151	0.337	1.6%	1.16 [0.60, 2.25]
KWMC_5.3.0	0.61	0.57	0.5%	1.84 [0.60, 5.62]
NHIMC_5.3.0	0.383	0.182	5.3%	1.47 [1.03, 2.10]
PNUH_5.3.0	0.921	0.992	0.2%	2.51 [0.36, 17.55]
WKUH_5.3.0	0.432	0.351	1.4%	1.54 [0.77, 3.06]
**Subtotal (95% CI)**			**13.6%**	**1.28 [1.02, 1.60]**
Heterogeneity: Tau² = 0.00; Chi² = 5.80, df = 7 (P = 0.56); I² = 0%				
Test for overall effect: Z = 2.14 (P = 0.03)				
**2.1.4 constipation**				
AUMC_5.3.0	0.245	0.159	7.0%	1.28 [0.94, 1.74]
DCMC_5.3.0	0.184	0.351	1.4%	1.20 [0.60, 2.39]
KDH_5.3.0	0.297	0.315	1.8%	1.35 [0.73, 2.50]
KHNMC_5.3.0	-0.025	0.318	1.7%	0.98 [0.52, 1.82]
KWMC_5.3.0	0.423	0.342	1.5%	1.53 [0.78, 2.98]
NHIMC_5.3.0	0.339	0.126	11.1%	1.40 [1.10, 1.80]
PNUH_5.3.0	0.071	0.4	1.1%	1.07 [0.49, 2.35]
WKUH_5.3.0	0.663	0.29	2.1%	1.94 [1.10, 3.43]
**Subtotal (95% CI)**			**27.8%**	**1.35 [1.15, 1.58]**
Heterogeneity: Tau² = 0.00; Chi² = 3.39, df = 7 (P = 0.85); I² = 0%				
Test for overall effect: Z = 3.77 (P = 0.0002)				
**Total (95% CI)**			**100.0%**	**1.34 [1.24, 1.46]**
Heterogeneity: Tau² = 0.00; Chi² = 20.61, df = 31 (P = 0.92); I² = 0%				
Test for overall effect: Z = 7.03 (P < 0.00001)				
Test for subgroup differences: Chi² = 0.49, df = 3 (P = 0.92), I² = 0%				

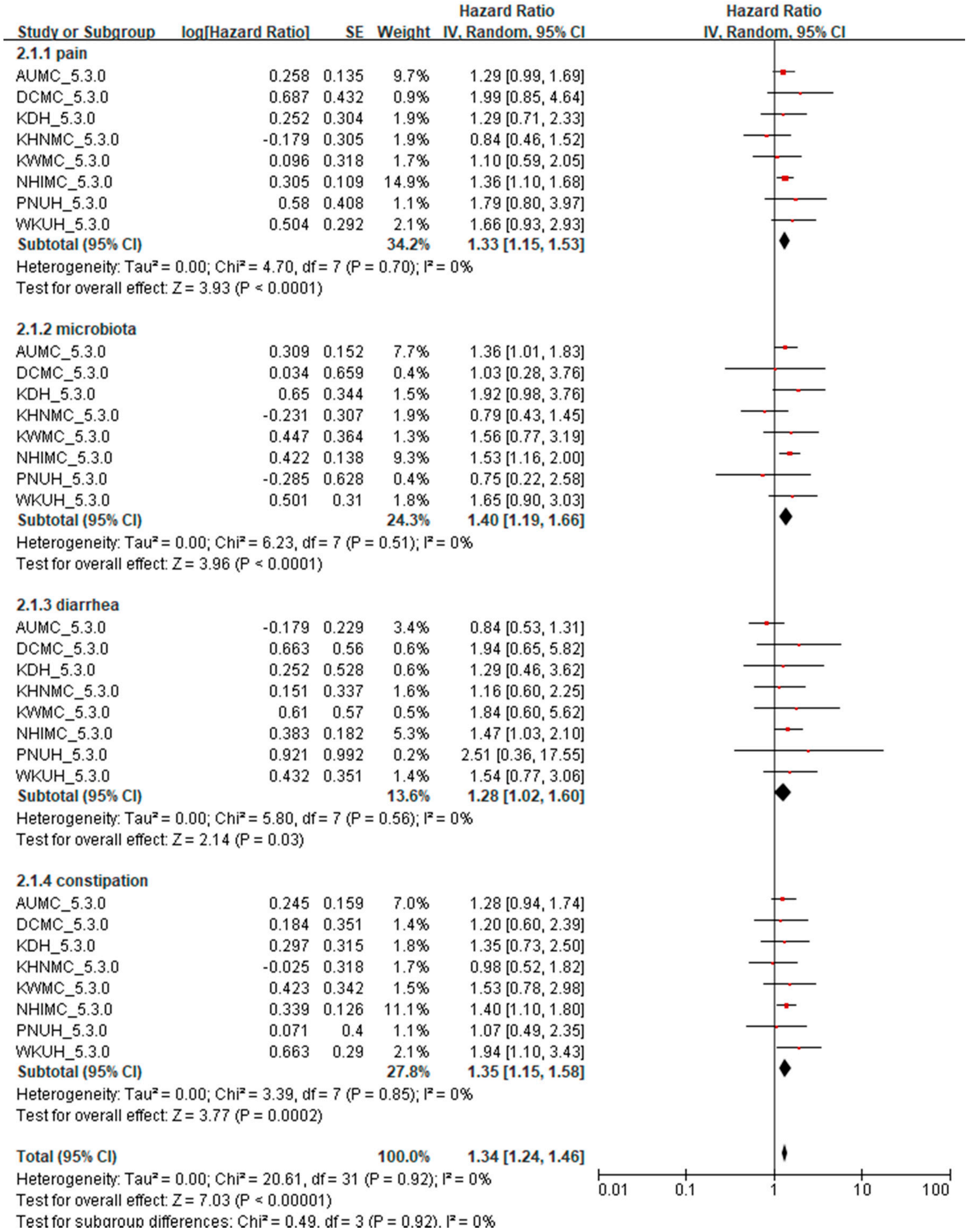

Supplement 3. Subgroup analysis for the risk of IBS treatment on osteoporotic fracture.

Study or Subgroup	log[Hazard Ratio]	SE	Weight	Hazard Ratio IV, Random, 95% CI
**2.2.1 pain**				
AUMC_5.3.0	-0.18	0.255	4.6%	0.84 [0.51, 1.38]
DCMC_5.3.0	-0.084	0.621	0.9%	0.92 [0.27, 3.11]
KDH_5.3.0	0.333	0.238	5.2%	1.40 [0.88, 2.22]
KHNMC_5.3.0	0.075	0.333	2.8%	1.08 [0.56, 2.07]
KWMC_5.3.0	-0.042	0.392	2.1%	0.96 [0.44, 2.07]
NHIMC_5.3.0	0.329	0.15	11.0%	1.39 [1.04, 1.86]
PNUH_5.3.0	-0.353	0.732	0.6%	0.70 [0.17, 2.95]
WKUH_5.3.0	0.367	0.424	1.8%	1.44 [0.63, 3.31]
**Subtotal (95% CI)**			**29.0%**	**1.21 [0.99, 1.46]**
Heterogeneity: Tau² = 0.00; Chi² = 4.71, df = 7 (P = 0.69); I² = 0%				
Test for overall effect: Z = 1.90 (P = 0.06)				
**2.2.2 microbiota**				
AUMC_5.3.0	-0.174	0.286	3.7%	0.84 [0.48, 1.47]
DCMC_5.3.0	-1.389	1.184	0.2%	0.25 [0.02, 2.54]
KDH_5.3.0	0.275	0.218	6.0%	1.32 [0.86, 2.02]
KHNMC_5.3.0	-0.328	0.352	2.5%	0.72 [0.36, 1.44]
KWMC_5.3.0	0.003	0.433	1.7%	1.00 [0.43, 2.34]
NHIMC_5.3.0	0.258	0.185	8.0%	1.29 [0.90, 1.86]
PNUH_5.3.0	-0.945	1.26	0.2%	0.39 [0.03, 4.59]
WKUH_5.3.0	0.393	0.39	2.1%	1.48 [0.69, 3.18]
**Subtotal (95% CI)**			**24.5%**	**1.12 [0.90, 1.39]**
Heterogeneity: Tau² = 0.00; Chi² = 6.64, df = 7 (P = 0.47); I² = 0%				
Test for overall effect: Z = 1.04 (P = 0.30)				
**2.2.3 diarrhea**				
AUMC_5.3.0	-0.357	0.384	2.2%	0.70 [0.33, 1.49]
DCMC_5.3.0	-1.051	0.831	0.5%	0.35 [0.07, 1.78]
KDH_5.3.0	-0.002	0.341	2.7%	1.00 [0.51, 1.95]
KHNMC_5.3.0	-1.018	0.428	1.8%	0.36 [0.16, 0.84]
KWMC_5.3.0	0.095	0.431	1.7%	1.10 [0.47, 2.56]
NHIMC_5.3.0	0.163	0.221	5.9%	1.18 [0.76, 1.82]
PNUH_5.3.0	-0.247	1.393	0.2%	0.78 [0.05, 11.98]
WKUH_5.3.0	-0.205	0.472	1.5%	0.81 [0.32, 2.05]
**Subtotal (95% CI)**			**16.3%**	**0.85 [0.63, 1.15]**
Heterogeneity: Tau² = 0.03; Chi² = 8.09, df = 7 (P = 0.33); I² = 13%				
Test for overall effect: Z = 1.04 (P = 0.30)				
**2.2.4 constipation**				
AUMC_5.3.0	-0.632	0.293	3.6%	0.53 [0.30, 0.94]
DCMC_5.3.0	-0.214	0.403	2.0%	0.81 [0.37, 1.78]
KDH_5.3.0	-0.02	0.203	6.8%	0.98 [0.66, 1.46]
KHNMC_5.3.0	-0.17	0.361	2.4%	0.84 [0.42, 1.71]
KWMC_5.3.0	-0.394	0.392	2.1%	0.67 [0.31, 1.45]
NHIMC_5.3.0	-0.118	0.16	10.0%	0.89 [0.65, 1.22]
PNUH_5.3.0	-0.654	0.694	0.7%	0.52 [0.13, 2.03]
WKUH_5.3.0	-0.333	0.345	2.6%	0.72 [0.36, 1.41]
**Subtotal (95% CI)**			**30.2%**	**0.81 [0.67, 0.98]**
Heterogeneity: Tau² = 0.00; Chi² = 4.05, df = 7 (P = 0.77); I² = 0%				
Test for overall effect: Z = 2.12 (P = 0.03)				
**Total (95% CI)**			**100.0%**	**0.99 [0.88, 1.11]**
Heterogeneity: Tau² = 0.01; Chi² = 33.53, df = 31 (P = 0.35); I² = 8%				
Test for overall effect: Z = 0.22 (P = 0.83)				
Test for subgroup differences: Chi² = 10.29, df = 3 (P = 0.02), I² = 70.8%				

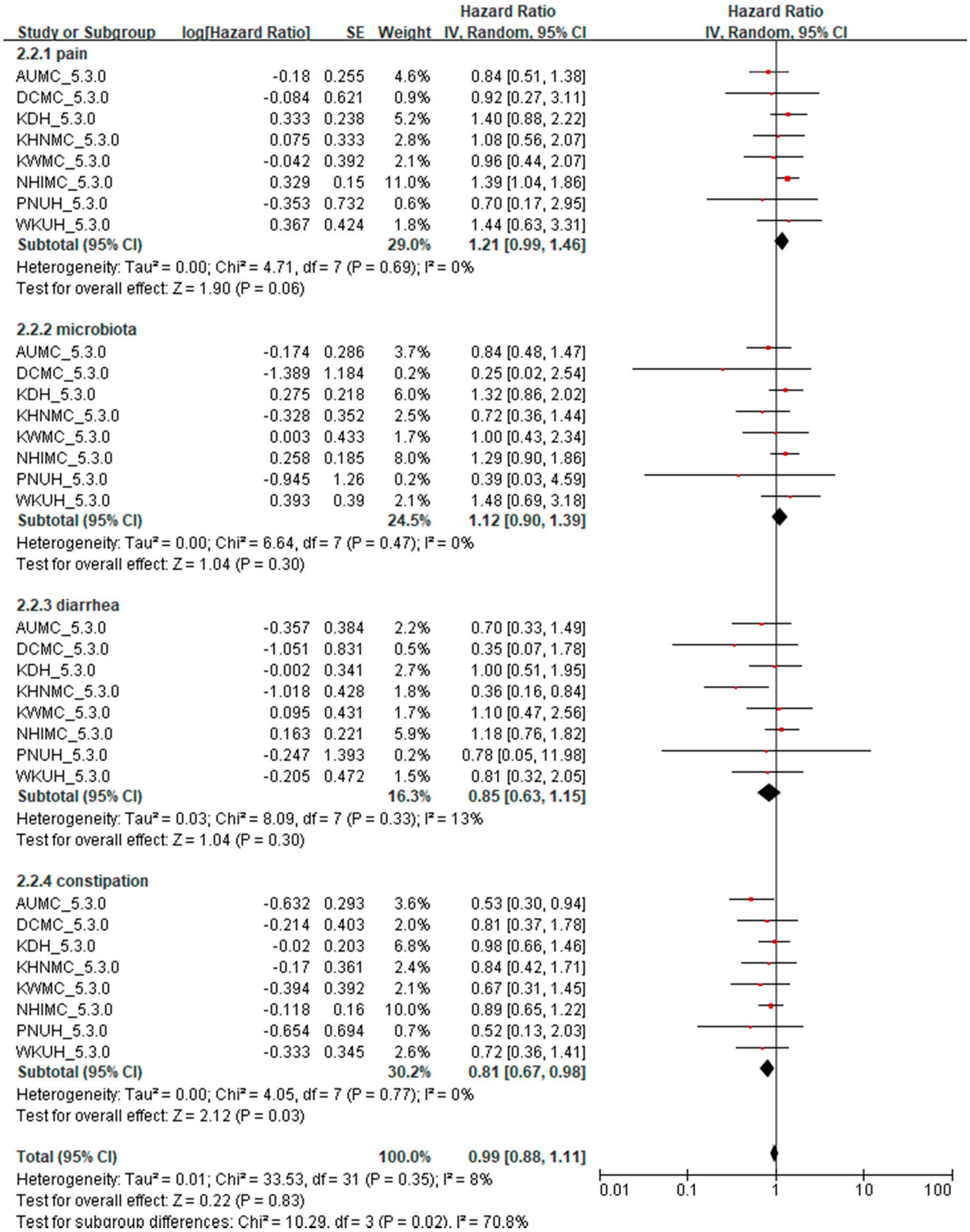

Supplement 4. The number of patients who were prescribed IBS treatment drugs.

Prescribed medications IBS			IBS			Non-IBS		p-value
			Patients	%		Patients	%	
Pain	Amitriptyline		174	0.62		5,228	0.83	<0.001
	Butylscopolamine		1,770	6.26		14,832	2.36	<0.001
	Cimetidine		2,408	8.52		42,455	6.77	<0.001
	Escitalopram		28	0.10		2,024	0.32	<0.001
	Fluoxetine		0	0.00		92	0.01	<0.001
	Imipramine		0	0.00		160	0.03	<0.001
	Mebeverine		40	0.14		11	0.00	<0.001
	Octylonium		1,270	4.49		750	0.12	<0.001
	Paroxetine		5	0.02		209	0.03	0.058
	Pinaverium		2,512	8.88		1,217	0.19	<0.001
	Sum of patients for pain		8,207	29.03		66,978	10.68	<0.001
Mcrobiota	Bacillus subtilis		2,294	8.11		15,417	2.46	<0.001
	Lactobacillus Acidophilus		994	3.52		7,214	1.15	<0.001
	Lactobacillus casei Rhamnosus		782	2.77		435	0.07	<0.001
	Lactobacillus Rhamnosus r0011		300	1.06		1,571	0.25	<0.001
	Rifaximin		330	1.17		53	0.01	<0.001
	Saccharomyces Boulardii		3,612	12.78		6,032	0.96	<0.001
	Sum of patients for microbiota		8,312	29.40		30,722	4.90	<0.001
Diarrhea	Loperamide		460	1.63		688	0.11	<0.001
	Nortriptyline		95	0.34		1,612	0.26	0.024
	Ondansetron		4	0.01		789	0.13	<0.001
	Palonosetron		5	0.02		325	0.05	<0.001
	Tianeptine		822	2.91		1,410	0.22	<0.001
	Sum of patients for diarrhea		1,386	4.90		4,824	0.77	<0.001
Constipation	Calcium polycarbophil		1,643	5.81		1,578	0.25	<0.001
	Carboxymethylcellulose		8	0.03		1,277	0.20	<0.001
	Cagnesium oxide		751	2.66		5,092	0.81	<0.001
	Polyethylene glycol 3350		3,330	11.78		6,933	1.11	<0.001
	Pyridostigmine		77	0.27		3,105	0.50	<0.001
	Tegaserod		462	1.63		15	0.00	<0.001
	Sum of patients for constipation		6,271	22.18		18,000	2.87	<0.001
Total		28,273	24,176	85.51	627,171	120,524	19.22	<0.001

Total information (in red) may have been overestimated because one patient has been prescribed more than one medication.

Supplement 5. Forest plot of meta-analysis who taken IBS treatment.

Study or Subgroup	log[HR]	SE	Weight	HR IV, Random, 95% CI
AUMC_5.3.0	0.041	0.083	18.6%	1.04 [0.89, 1.23]
DCMC_5.3.0	0.371	0.155	6.0%	1.45 [1.07, 1.96]
KDH_5.3.0	-0.052	0.105	12.4%	0.95 [0.77, 1.17]
KHNMC_5.3.0	0.035	0.128	8.6%	1.04 [0.81, 1.33]
KWMC_5.3.0	0.265	0.141	7.2%	1.30 [0.99, 1.72]
NHIMC_5.3.0	0.081	0.053	37.3%	1.08 [0.98, 1.20]
PNUH_5.3.0	0.199	0.205	3.5%	1.22 [0.82, 1.82]
WKUH_5.3.0	0.003	0.15	6.4%	1.00 [0.75, 1.35]
**Total (95% CI)**			**100.0%**	**1.09 [1.01, 1.17]**

Heterogeneity: Tau² = 0.00; Chi² = 7.77, df = 7 (P = 0.35); I² = 10%
Test for overall effect: Z = 2.13 (P = 0.03)

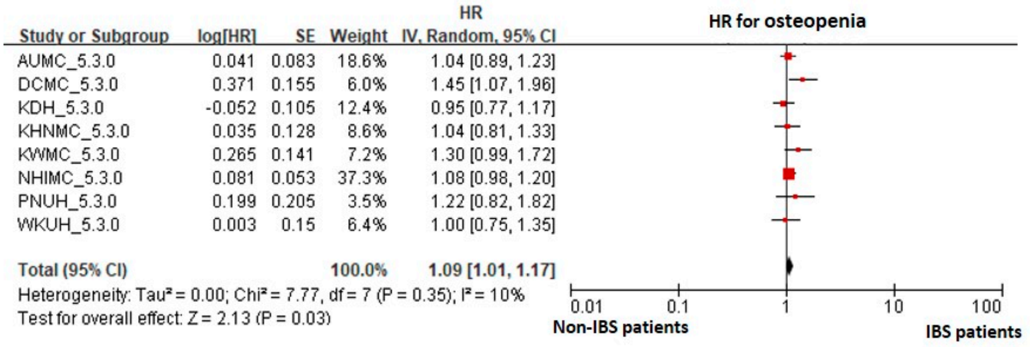

Study or Subgroup	log[Hazard Ratio]	SE	Weight	Hazard Ratio IV, Random, 95% CI
**2.3.1 pain**				
AUMC_5.3.0	0.068	0.086	5.2%	1.07 [0.90, 1.27]
DCMC_5.3.0	0.34	0.238	1.6%	1.40 [0.88, 2.24]
KDH_5.3.0	0.08	0.125	3.7%	1.08 [0.85, 1.38]
KHNMC_5.3.0	0.12	0.132	3.5%	1.13 [0.87, 1.46]
KWMC_5.3.0	0.219	0.138	3.3%	1.24 [0.95, 1.63]
NHIMC_5.3.0	0.095	0.055	6.5%	1.10 [0.99, 1.22]
PNUH_5.3.0	0.323	0.226	1.7%	1.38 [0.89, 2.15]
WKUH_5.3.0	0.252	0.188	2.2%	1.29 [0.89, 1.86]
**Subtotal (95% CI)**			**27.7%**	**1.13 [1.05, 1.21]**
Heterogeneity: Tau² = 0.00; Chi² = 3.34, df = 7 (P = 0.85); I² = 0%				
Test for overall effect: Z = 3.15 (P = 0.002)				
**2.3.2 microbiota**				
AUMC_5.3.0	0.012	0.098	4.7%	1.01 [0.84, 1.23]
DCMC_5.3.0	-0.189	0.263	1.3%	0.83 [0.49, 1.39]
KDH_5.3.0	0.067	0.116	4.0%	1.07 [0.85, 1.34]
KHNMC_5.3.0	0.032	0.152	3.0%	1.03 [0.77, 1.39]
KWMC_5.3.0	0.228	0.156	2.9%	1.26 [0.93, 1.71]
NHIMC_5.3.0	0.025	0.072	5.8%	1.03 [0.89, 1.18]
PNUH_5.3.0	0.402	0.31	1.0%	1.49 [0.81, 2.74]
WKUH_5.3.0	-0.072	0.187	2.2%	0.93 [0.64, 1.34]
**Subtotal (95% CI)**			**24.8%**	**1.04 [0.96, 1.14]**
Heterogeneity: Tau² = 0.00; Chi² = 4.11, df = 7 (P = 0.77); I² = 0%				
Test for overall effect: Z = 0.94 (P = 0.35)				
**2.3.3 diarrhea**				
AUMC_5.3.0	-0.258	0.138	3.3%	0.77 [0.59, 1.01]
DCMC_5.3.0	-0.105	0.257	1.4%	0.90 [0.54, 1.49]
KDH_5.3.0	-0.386	0.179	2.4%	0.68 [0.48, 0.97]
KHNMC_5.3.0	-0.244	0.166	2.6%	0.78 [0.57, 1.08]
KWMC_5.3.0	-0.397	0.203	2.0%	0.67 [0.45, 1.00]
NHIMC_5.3.0	-0.029	0.093	4.9%	0.97 [0.81, 1.17]
PNUH_5.3.0	0.693	0.386	0.7%	2.00 [0.94, 4.26]
WKUH_5.3.0	-0.351	0.21	1.9%	0.70 [0.47, 1.06]
**Subtotal (95% CI)**			**19.2%**	**0.82 [0.70, 0.96]**
Heterogeneity: Tau² = 0.02; Chi² = 11.41, df = 7 (P = 0.12); I² = 39%				
Test for overall effect: Z = 2.43 (P = 0.02)				
**2.3.4 constipation**				
AUMC_5.3.0	-0.206	0.098	4.7%	0.81 [0.67, 0.99]
DCMC_5.3.0	0.129	0.171	2.5%	1.14 [0.81, 1.59]
KDH_5.3.0	-0.203	0.113	4.1%	0.82 [0.65, 1.02]
KHNMC_5.3.0	-0.183	0.146	3.1%	0.83 [0.63, 1.11]
KWMC_5.3.0	0.165	0.149	3.0%	1.18 [0.88, 1.58]
NHIMC_5.3.0	-0.137	0.065	6.1%	0.87 [0.77, 0.99]
PNUH_5.3.0	0.236	0.201	2.0%	1.27 [0.85, 1.88]
WKUH_5.3.0	-0.286	0.158	2.8%	0.75 [0.55, 1.02]
**Subtotal (95% CI)**			**28.4%**	**0.91 [0.81, 1.01]**
Heterogeneity: Tau² = 0.01; Chi² = 11.58, df = 7 (P = 0.12); I² = 40%				
Test for overall effect: Z = 1.75 (P = 0.08)				
**Total (95% CI)**			**100.0%**	**0.99 [0.93, 1.05]**

Heterogeneity: Tau² = 0.01; Chi² = 58.58, df = 31 (P = 0.002); I² = 47%
Test for overall effect: Z = 0.34 (P = 0.73)
Test for subgroup differences: Chi² = 18.59, df = 3 (P = 0.0003), I² = 83.9%

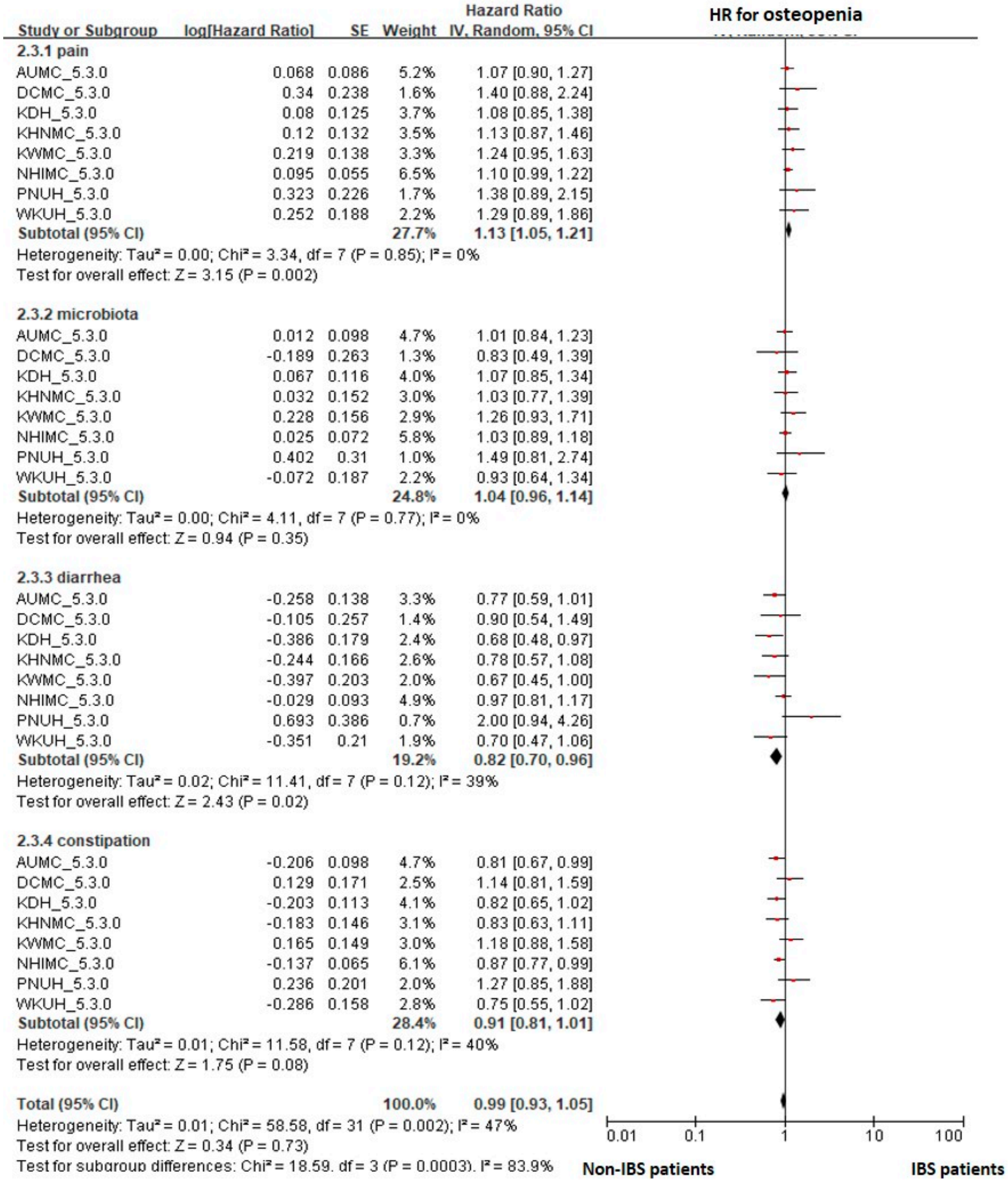

Supplement: Supplementary file 1 [file jcm-10-02044-s001.zip › jcm-1143233-supplementary.pdf]
